# Organic Crosslinked Polymer-Derived N/O-Doped Porous Carbons for High-Performance Supercapacitor

**DOI:** 10.3390/nano12132186

**Published:** 2022-06-25

**Authors:** Jianhao Lao, Yao Lu, Songwen Fang, Fen Xu, Lixian Sun, Yu Wang, Tianhao Zhou, Lumin Liao, Yanxun Guan, Xueying Wei, Chenchen Zhang, Yukai Yang, Yongpeng Xia, Yumei Luo, Yongjin Zou, Hailiang Chu, Huanzhi Zhang, Yong Luo, Yanling Zhu

**Affiliations:** 1 Guangxi Key Laboratory of Information Materials, Guangxi Collaborative Innovation Center for Structure and Properties for New Energy and Materials, School of Material Science and Engineering, Guilin University of Electronic Technology, Guilin 541004, China; ljh408888@163.com (J.L.); 18677367876@163.com (Y.L.); 1810201010@mails.guet.edu.cn (S.F.); ywang506x@163.com (Y.W.); zhoutianhao233@gmail.com (T.Z.); llm904691049@163.com (L.L.); gyx112405@163.com (Y.G.); Zhang_linba_3760@163.com (C.Z.); yangyukai0530@gmail.com (Y.Y.); ypxia@guet.edu.cn (Y.X.); luoym@guet.edu.cn (Y.L.); zouy@guet.edu.cn (Y.Z.); chuhailiang@guet.edu.cn (H.C.); zhanghuanzhi@guet.edu.cn (H.Z.); 15620323072@139.com (Y.L.); zyl9352021@163.com (Y.Z.); 2 School of Electronic Engineering and Automation, Guilin University of Electronic Technology, Guilin 541004, China; 3 School of Architecture and Transportation Engineering, Guilin University of Electronic Technology, Guilin 541004, China

**Keywords:** supercapacitor, organic crosslinked polymer, porous carbon, electrochemistry

## Abstract

Supercapacitors, as a new type of green electrical energy storage device, are a potential solution to environmental problems created by economic development and the excessive use of fossil energy resources. In this work, nitrogen/oxygen (N/O)-doped porous carbon materials for high-performance supercapacitors are fabricated by calcining and activating an organic crosslinked polymer prepared using polyethylene glycol, hydroxypropyl methylcellulose, and 4,4-diphenylmethane diisocyanate. The porous carbon exhibits a large specific surface area (1589 m^2^·g^−1^) and high electrochemical performance, thanks to the network structure and rich N/O content in the organic crosslinked polymer. The optimized porous carbon material (C_OCLP-4.5_), obtained by adjusting the raw material ratio of the organic crosslinked polymer, exhibits a high specific capacitance (522 F·g^−1^ at 0.5 A·g^−1^), good rate capability (319 F·g^−1^ at 20 A·g^−1^), and outstanding stability (83% retention after 5000 cycles) in a three-electrode system. Furthermore, an energy density of 18.04 Wh·kg^−1^ is obtained at a power density of 200.0 W·kg^−1^ in a two-electrode system. This study demonstrates that organic crosslinked polymer-derived porous carbon electrode materials have good energy storage potential.

## 1. Introduction

Solutions to environmental problems, owing to economic development and the excessive use of fossil energy resources, are urgently being sought [1]. Supercapacitors, as a new type of green electrical energy storage device, have drawn increasing attention, owing to their high power density, fast charging/discharging, excellent reversibility, long life cycle, and environmental friendliness [2,3,4].

Theoretical research on and practical applications of supercapacitors have significantly progressed; however, insufficient energy density and high cost are still challenges requiring resolution [5,6,7]. Electrode materials, which can be divided into carbon materials [8,9], metal oxides [10,11], and conductive polymers [12,13], play an important role as core components in supercapacitors and are a key step in solving the existing problems. Among them, carbon materials are the most widely used electrode materials because of their high specific surface area, and good electrical conductivity and chemical stability [14,15,16]. Studies have shown that doping heteroatoms in a carbon-based framework increases the specific capacitance of carbon materials. On the one hand, it can improve the infiltration area between the electrode material and the electrolyte; on the other hand, the heterogeneous atoms can introduce pseudocapacitance during the charging/discharging process, further enhancing the electrochemical performance [17].

Nitrogen doping has been demonstrated to be an effective way to improve the wettability and conductivity of carbon materials and can also provide additional pseudocapacitance for supercapacitors. Generally, nitrogen-doped carbon materials can be prepared using two synthetic strategies, namely by the pyrolysis of nitrogen-containing precursors, such as biomass [18], synthetic polymers [19], small molecules [20], and ionic liquids [21], or by the chemical or thermal modification of premade carbon materials with reagents/gases containing nitrogen atoms [22]. Zhang et al. [23] used urea as a nitrogen-containing precursor and KOH as the activator to prepare a carbon material with an appropriate amount of N doping, which yielded a nitrogen-doped carbon material with a porous structure and large specific surface area. They also found that the capacitance of the carbon material reached up to 446.0 F·g^−1^ at 0.5 A·g^−1^ in a three-electrode system. The symmetrical supercapacitor device assembled with this nitrogen-doped carbon also displayed good performance, with an energy density of 16.3 Wh·kg^−1^ at a power density of 348.3 W·kg^−1^.

Organic crosslinked polymers are mainly composed of elements, such as carbon, nitrogen, oxygen, and hydrogen, which have the characteristics of a network structure. Porous carbon materials prepared using such polymers had a high heteroatom content, specific surface area, and outstanding electrochemical properties [24]. In particular, the structure of organic crosslinked polymers can be adjusted by changing the ratio of raw materials during the synthesis process. Zou et al. [25] prepared a new type of heteroatom-doped porous carbon material with a high specific surface area by carbonizing and activating polyphosphazenes, which exhibited a specific capacitance of 438 F·g^−1^ at a current density of 0.5 A·g^−1^ in a three-electrode system. Chen et al. [26] prepared a porous carbon material by calcining hypercrosslinked polymer (poly (vinylbenzyl chloride-co-divinylbenzene)), which exhibited a specific capacitance of 455 F·g^−1^ at a current density of 0.5 A·g^−1^.

In this work, nitrogen/oxygen(N/O)-doped carbon-based porous materials were fabricated by carbonizing and activating an organic crosslinked polymer with a network structure. The organic crosslinked polymer was synthesized using polyethylene glycol (PEG 6000), hydroxypropyl methylcellulose (HPMC), and 4,4-diphenylmethane diisocyanate (MDI). The carbon material obtained by optimizing the ratio of the raw materials had a large specific surface area (1589 m^2^·g^−1^) and a high specific capacitance of 522 F·g^−1^ at a current density of 0.5 A·g^−1^. Furthermore, its energy density reached 18.04 Wh·kg^−1^ at a power density of 200.0 W·kg^−1^ in a two-electrode system using 1 M Na_2_SO_4_ as the electrolyte. Mechanistic studies showed that the high electrochemical performance of the obtained carbon was attributed to the network structure and rich N/O content of the crosslinked polymer. Hence, the preparation method for porous carbon materials proposed in this study provides a new approach for the research and development of electrode materials.

## 2. Materials and Methods

### 2.1. Materials

Polyethylene glycol (PEG, Mw = 6000), 4,4-diphenylmethane diisocyanate (MDI, analytical grade), hydroxypropyl methylcellulose (HPMC, Mw = 10,000), polytetrafluoroethylene (PTFE), and *N*, *N*-dimethylformamide (DMF) were purchased from Aladdin. Analytical-grade potassium hydroxide (KOH) and acetylene black were obtained from Xilong Science Co., Ltd. (Shantou, China). None of the purchased reagents were purified before use. All aqueous solutions were prepared using ultrapure water (deionized water, resistance 18 MΩ cm^−1^).

### 2.2. Synthesis of Organic Crosslinked Polymers

**Figure 1:**
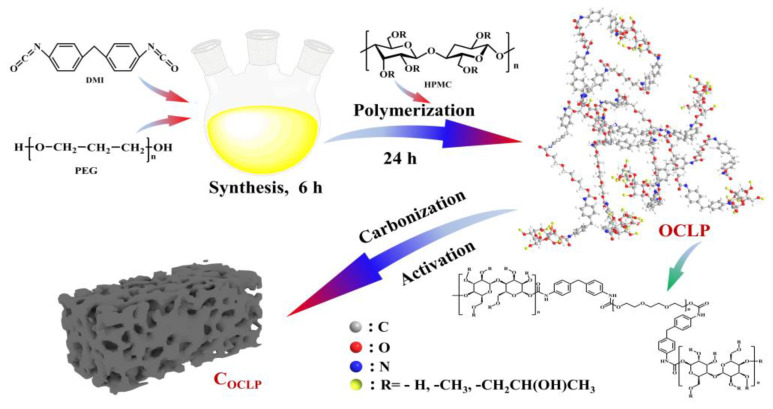
Schematic illustration of the one-pot method for the preparation of organic crosslinked polymer-derived porous carbon.

The organic crosslinked polymers were prepared by a one-pot method, which is a minor modification based on our previous report [27]. Briefly, PEG 6000 (12.0 g), MDI (1.0 g), and a certain amount of HPMC were stirred in a three-neck flask containing DMF (80 mL) under argon gas and an oil bath with a constant temperature of 75 °C. The organic crosslinked polymer obtained after 30 h of condensation reflux is referred to as OCLP. The mass of HPMC was 3.5, 4.5, and 5.0 g; therefore, the corresponding organic crosslinked polymers were named as OCLP_3.5_, OCLP_4.5_ and OCLP_5.0_, respectively. Figure 1 presents a flowchart of the one-pot method for the preparation of organic crosslinked polymers.

### 2.3. Preparation of Porous Carbon Materials

The prepared OCLPs were directly carbonized by heating them in a tube furnace at 500 °C for 2 h under a N_2_ atmosphere at a heating rate of 5 °C/min. The resulting carbon precursors were homogeneously ground with KOH in a mass ratio of 1.0:3.0, then calcined in a tube furnace at 600 °C under a N_2_ atmosphere for 2 h. The calcined products were stirred with a 1 M hydrochloric acid solution for 2 h, followed by washing with distilled water and anhydrous ethanol sequentially until the filtrate was neutral. The obtained residues were dried in a blast oven at 80 °C for 24 h to obtain porous carbon materials, which were named as C_OCLP-3.5_, C_OCLP-4.5_, and C_OCLP-5.0_, respectively.

### 2.4. Characterization

Fourier transform infrared (FTIR) spectroscopy was performed on the samples using a Thermo Fisher (Waltham, MA, USA) Nicolet 6700 spectrometer with KBr pellets. A powder X-ray diffractometer (XRD; D8 Advance Bruker, Billerica, MA, USA) operating at 40 kV and 40 mA with Cu Kα radiation (λ = 0.15406 nm) in the 2θ range of 5–90° with 0.01° step increments was used to analyze the microstructure of the materials. The chemical structure and graphitization of the samples were further characterized using Raman spectroscopy (Horiba JY, Palaiseau, France) at an excitation wavelength of 532 nm. The surface micromorphology of the samples was characterized using scanning electron microscopy (SEM; SU8010, HITACHI, Tokyo, Japan) and transmission electron microscopy (TEM; Tecnai G2 F20, FEI Company, Hillsboro, OR, USA), and elemental analysis was performed using energy-dispersive X-ray spectroscopy (EDS). The specific surface area and pore structure characteristics of the samples were characterized using a nitrogen adsorption–desorption analyzer (ASIQM0002-4, Quantachrome, Boynton Beach, Florida, USA) at −196 °C. Surface element analysis was performed using X-ray photoelectron spectroscopy (XPS; Thermo Scientific Escalab 250Xi, Waltham, MA, USA).

### 2.5. Electrochemical Measurements

The electrochemical performance of the samples, including galvanostatic charge–discharge (GCD), cyclic voltammetry (CV), and electrochemical impedance (EIS), was measured using a CHI 660E instrument in a three-electrode system. A slurry mixture of carbon material (C_OCLP_), acetylene black, and PTFE in a weight ratio of 8:1:1 was applied to nickel foam (2 cm × 2 cm) as the working electrode; platinum and Hg/HgO electrodes were used as the counter and reference electrodes, respectively, in the three-electrode system. The voltage was set to −1–0 V and the electrolyte was 6 M KOH. A symmetric supercapacitor was built for a two-electrode system using the C_OCLP_, a 1 M Na_2_SO_4_ electrolyte, and a voltage range of 0–1.6 V.

For the three-electrode and two-electrode systems, the weight-specific capacitances (F·g^−1^) of the electrode material were calculated based on the GCD curves using Equations (1) and (2), respectively.

(1) $$C_{g}=\frac{I\Delta t}{m\Delta V}$$

(2) $$C_{g}=\frac{2I\Delta t}{m\Delta V}$$

where *I* (A), Δ*t* (s), Δ*V* (mV), and *m* (g) represent the discharge current, discharge time, discharge voltage range, and mass of the active material of a single electrode, respectively.

The energy density (*E_cell_*) and power density (*P_cell_*) of the symmetrical supercapacitor were calculated using Equations (3) and (4), respectively.

(3) $$E_{cell}=\frac{C_{g}\;\Delta V^{2}}{8\times 3.6}$$

(4) $$P_{cell}=\frac{3600\;E_{cell}}{\Delta t}$$

where *C*_g_ is obtained from Equation (2), Δ*V* is the working voltage of the discharge, and Δ*t* is the discharge time.

## 3. Results and Discussion

### 3.1. Structural and Morphological Characterization

**Figure 2:**
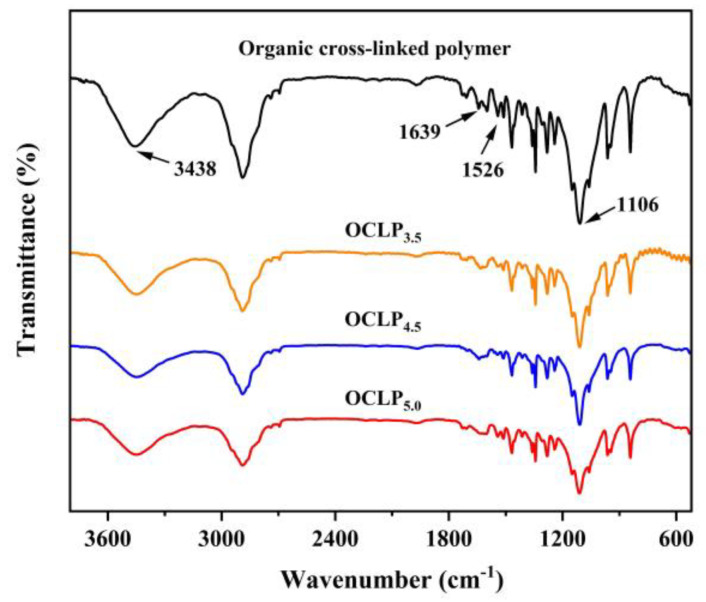
FTIR spectra of the OCLPs and organic crosslinked polymer [27].

Figure 2 shows the FTIR spectra of the samples, which indicates that the characteristic absorption peaks for the OCLPs (OCLP_3.5_, OCLP_4.5_, and OCLP_5.0_) are similar. The peaks around 3438, 1639, 1526, and 1106 cm^−1^ correspond to the stretching vibration absorption peaks of the –OH, C=O, C–N, and C–O groups, respectively, which is consistent with the organic crosslinked polymer [27]. The above results illustrate that the OCLPs are a type of organic crosslinked polymer.

**Figure 3:**
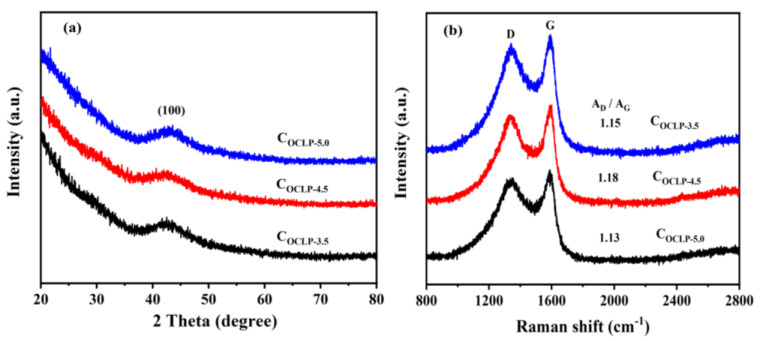
(**a**) XRD patterns of the C_OCLPs_ and (**b**) Raman spectra of the C_OCLPs_.

The C_OCLPs_ obtained from the OCLPs were characterized using XRD and Raman spectroscopy. Figure 3a summarizes the XRD spectra of the C_OCLP-3.5_, C_OCLP-4.5_, and C_OCLP-5.0_, showing that all the C_OCLPs_ exhibit obvious diffraction peaks at 43°, corresponding to the (100) crystal planes of the graphite structure. The results indicate that C_OCLP-3.5_, C_OCLP-4.5,_ and C_OCLP-5.0_ have amorphous graphite structures [28,29]. The diffraction peak intensity of the (100) lattice plane for C_OCLP-4.5_ is the weakest, demonstrating that C_OCLP-4.5_ has the highest structural disorder [30]. Figure 3b shows that there are two characteristic peaks at 1343 and 1594 cm^−1^, corresponding to the D and G peaks of graphite, respectively. The ratio of the areas of the D peak to the G peak (*A_D_/A_G_*) reflects the order degree of the C_OCLP_ structure [31]. The calculated ratios for C_OCLP-3.5_, C_OCLP-4.5_, and C_OCLP-5.0_ are 1.15: 1, 1.18: 1, and 1.13: 1, respectively. This result also illustrates that C_OCLP-4.5_ has more defects because the D peak represents a defect peak caused by the low symmetry or irregularity of the carbon material [32].

**Figure 4:**
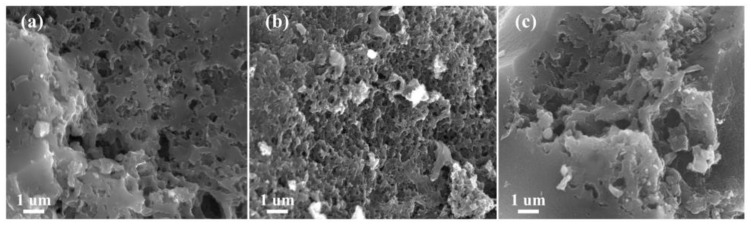
SEM images of samples, (**a**) C_OCLP-3.5_; (**b**) C_OCLP-4.5_; (**c**) C_OCLP-5.0_.

The surface morphologies of C_OCLP-3.5_, C_OCLP-4.5_, and C_OCLP-5.0_ were characterized using SEM, as shown in Figure 4. Figure 4 indicates that the three C_OCLPs_ are all porous and present a three-dimensional network structure. The number of pores in the C_OCLP_ increases with an increase in the amount of HPMC; however, when the HPMC content is increased to 5.0 g, the pore structure is only partially formed, and the number of pores decreases. The result demonstrates that the pore structure of C_OCLP-4.5_ was excellent. Generally, an abundant number of pores can significantly increase the specific surface area of C_OCLPs_, thereby providing more storage sites and transport channels for electrolyte ions. This is beneficial for improving the electrochemical performance [33].

**Figure 5:**
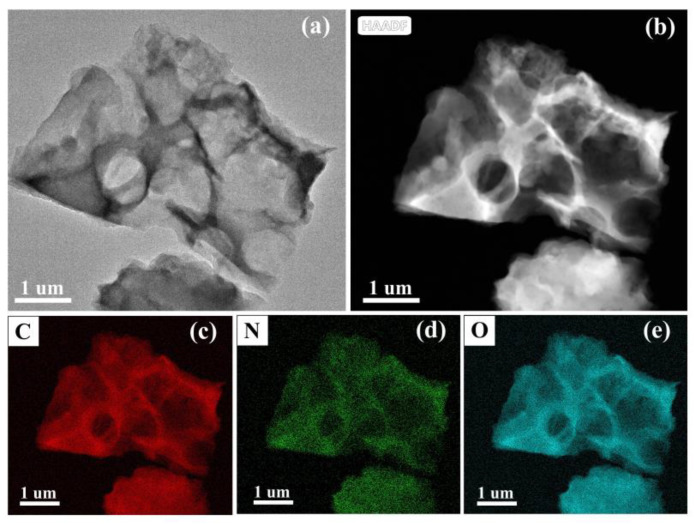
(**a**) TEM and (**b**–**e**) EDS images of C_OCLP-4.5_.

Additionally, Figure 5a further demonstrates that C_OCLP-4.5_ is a porous C_OCLP_. When C_OCLP-4.5_ is used as the electrode material, these disordered microporous structures can provide sufficient active sites for charge storage [34]. Figure 5b–e are element distribution diagrams obtained from the EDS analysis of C_OCLP-4.5_, showing that carbon, nitrogen, and oxygen were uniformly distributed in the carbon framework. Abundant nitrogen and oxygen can introduce pseudocapacitance and enhance the capacitance performance of the electrode material.

**Figure 6:**
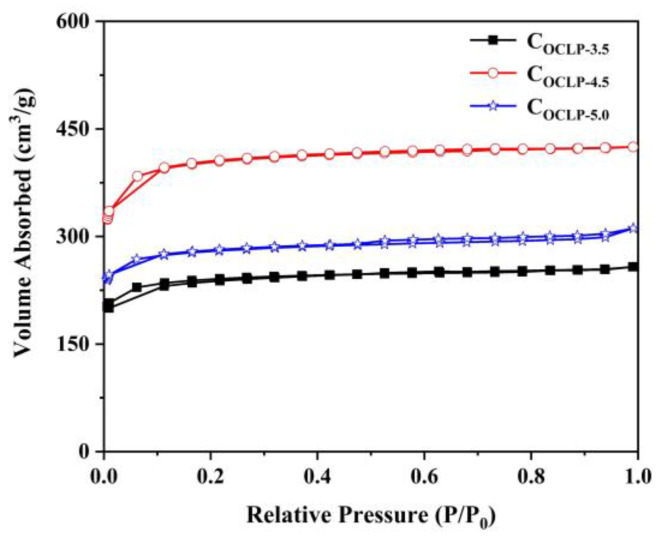
N_2_ desorption/adsorption isotherm curves of C_OCLPs_.

The C_OCLPs_ were subjected to N_2_ adsorption–desorption measurements to explore the pore characteristics. Figure 6 shows that all the C_OCLPs_ exhibit obvious type I isotherm characteristics, indicating that these samples are rich in micropores [35]. Table 1 summarizes the pore structure characteristics of the C_OCLPs_, showing that the specific surface area and pore volume of these samples are mainly provided by the micropores and mesopores. Among the three samples, C_OCLP-4.5_ has the largest specific surface area (1589 m^2^·g^−1^) and the highest pore volume (0.657 cm^3^·g^−1^), which further confirm that C_OCLP-4.5_ has the best pore structure. Numerous studies have demonstrated that the large specific surface area and rich pore structure of porous carbon material can greatly promote the storage and rapid migration of ions, resulting in the excellent specific capacitance performance of supercapacitors [36,37]. The aqueous electrolytes currently used in supercapacitors are mainly sulfuric acid (H_2_SO_4_, acidic), KOH (alkaline), and sodium sulfate (Na_2_SO_4_, neutral). The electrolyte ions in these electrolytes mainly exist as hydrated ions (H^+^, K^+^, OH^−^, Na^+^, and ${\mathrm{SO}}_{4}^{2-}$). Based on Table 1, it can be found that the C_OCLPs_ obtained can meet the fast migration requirements of these electrolyte ions, thereby significantly improving the conductivity of carbon-based electrodes and enhancing their electrochemical performance.

**Figure 7:**
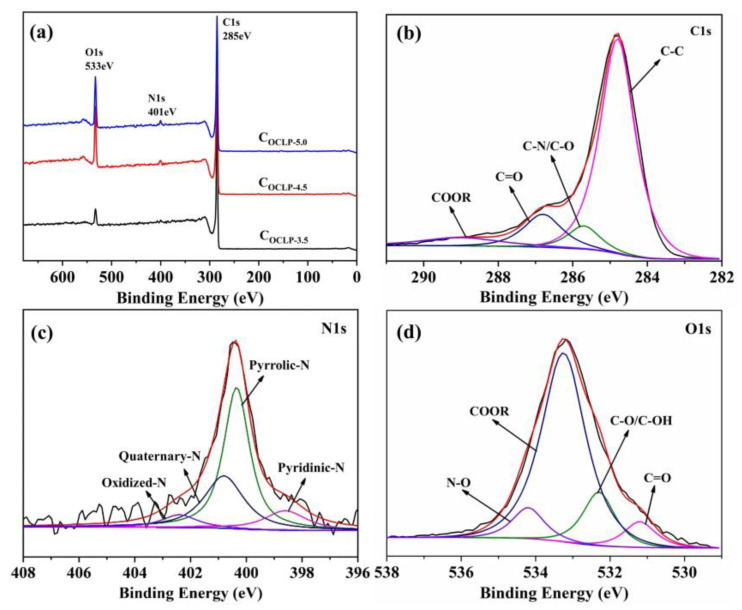
(**a**) XPS spectra of the C_OCLPs_; (**b**–**d**) high resolution of C 1s, N 1s and O 1s of C_OCLP-4.5_.

Further analysis of the surface electronic states and elemental compositions of the C_OCLPs_ samples was performed using XPS. Figure 7a shows that there are three peaks in the spectra of all the samples. The binding energies of the three peaks are 285, 400, and 532 eV, corresponding to C 1s, N 1s, and O 1s, respectively. The results also prove that carbon, nitrogen, and oxygen are present in the three samples. Table 2 lists the surface element contents of the three samples. These samples are mainly a carbon-based framework with oxygen and nitrogen. Fine analyses of the C 1s, N 1s, and O 1s spectra of C_OCLP-4.5_ are performed using the peak differentiation fitting method, as shown in Figure 7b–d. The C 1s spectrum (Figure 7b) can be matched by four peaks at 284.8, 285.7, 286.8, and 289.0 eV, corresponding to the C–C, C–N, C–O, and COOR groups, respectively [38]. The N 1s spectrum, shown in Figure 7c, is deconvoluted into four peaks of 398.8, 400.3, 400.8, and 402.4 eV, corresponding to pyridinic-N (N-6) (11.70%), pyrrolic-N (N-5) (52.13%), quaternary-N (N–Q) (29.79%), and oxidized N (N–X) (6.38%), respectively. In particular, the pyridinic-N and pyrrolic-N contents reach 63.83%. A high content of N-6 and N-5 is beneficial for introducing pseudo-capacitance and providing electrochemically active sites and quaternary nitrogen (N–Q) can effectively improve the conductivity of C_OCLPs_ and promote electron transfer in the carbon matrix [35,39]. The deconvoluted O 1s peak displayed four peaks at 531.2, 532.3, 533.3, and 534.2 eV, representing the oxygen atoms in the C=O, C–O/C–OH, COOR, and N–O groups, respectively (shown in Figure 7d) [35,39]. According to a previous report [40], the oxygen groups are evenly distributed in the carbon framework, which can improve the interfacial tension between the carbon-based porous material and electrolyte to reduce the interfacial resistance.

### 3.2. Electrochemistry Measurements

**Figure 8:**
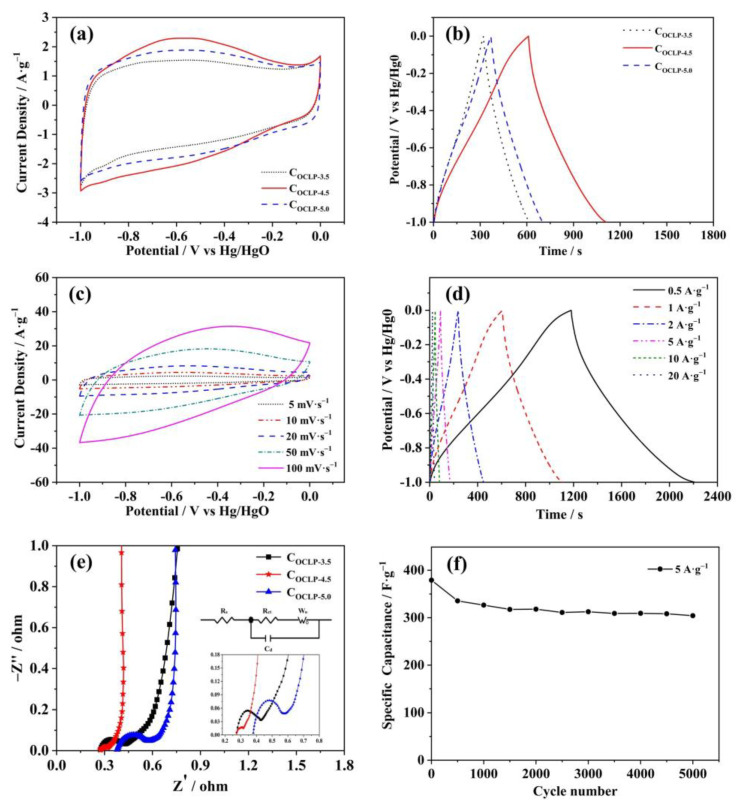
(**a**) CV curves of C_OCLPs_ at a scan rate of 5 mV·s^−1^; (**b**) GCD curves of C_OCLPs_ at a current density of 1 A·g^−1^; (**c**) CV curves of C_OCLP-4.5_ at different scan rates; (**d**) GCD curves of C_OCLP-4.5_ at different current densities; (**e**) Nyquist plots of the C_OCLPs_; (**f**) stable cyclic performance of C_OCLP-4.5_.

The electrochemical performances of the electrode materials were evaluated using a three-electrode system. Figure 8a shows the CV plots of the different C_OCLPs_ (C_OCLP-3.5_, C_OCLP-4.5_, and C_OCLP-5.0_) at a sweep rate of 5 mV·s^−1^. All the samples display a typical rectangular shape, indicating that the capacitive behavior of these materials is mainly electric double-layer capacitance. Concurrently, these curves have a broad peak in the voltage window of −0.8 to −0.3 V, which is caused by the oxidation–reduction reaction of nitrogen and oxygen atoms contained in these samples during the charge and discharge process. Moreover, the pseudo-capacitance introduced by the redox reaction can significantly increase the specific capacitance of carbon electrodes. The C_OCLP-4.5_ sample exhibits the largest encircled area of the CV curve among the three samples, which also illustrates that C_OCLP-4.5_ has the highest specific capacitance. Figure 8b shows the constant GCD curves for the C_OCLPs_ at a current density of 1 A·g^−1^. The GCD curves for the three samples are all quasi-isosceles triangle shapes, indicating that the capacitance is mainly electric double-layer capacitance (EDLC), and the slight deformation is attributed to the existence of pseudo-capacitance. According to Equation (1), the specific capacitances of C_OCLP-3.5_, C_OCLP-4.5_, and C_OCLP-5.0_ at a current density of 1 A·g^−1^ are 302, 503, and 330 F·g^−1^, respectively. This result shows that the specific capacitance of C_OCLP-4.5_ is the largest, owing to its large specific surface area (1589 m^2^·g^−1^) and pore volume (0.657 cm^3^·g^−1^). Figure 8c presents the CV curves for C_OCLP-4.5_ at different scanning rates. It reveals that the C_OCLP-4.5_ still maintains a quasi-rectangular shape at scan rates of 5–50 mV·s^−1^, indicating that the good pore structure of C_OCLP-4.5_ enables the rapid migration of electrolyte ions to result in its good rate capability. Figure 8d presents the GCD curves for C_OCLP-4.5_ at current densities of 0.5–20 A g^−1^, showing that the GCD curve does not exhibit a significant IR drop at a high current density of 20 A·g^−1^. Therefore, it demonstrates that the C_OCLP-4.5_ has a high conductivity, good rate capability, and electrochemical reversibility. The specific capacitances are calculated as 522, 503, 432, 396, 363, and 319 F·g^−1^ at current densities of 0.5, 1, 2, 5, 10, and 20 A·g^−1^, respectively. Comparing the electrochemical performance of C_OCLP-4.5_ with that of the references, the result is listed in Table 3. According to Table 3, the electrochemical performance of C_OCLP-4.5_ is better than that of other electroactive materials reported in the literature. This is attributed to the unique network structure and rich N/O content of the crosslinked polymer fabricated in this study.

**Figure 9:**
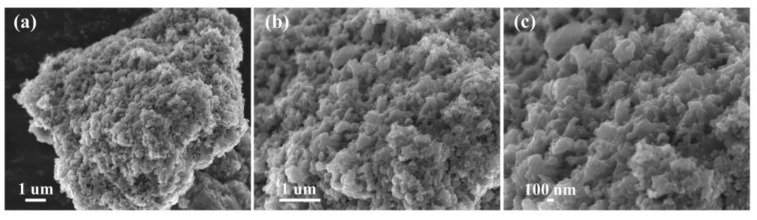
(**a**–**c**) SEM images of C_OCLP-4.5_ with different multiples after 5000 cycles.

Figure 8e presents the EIS curves for C_OCLPs_ and the equivalent circuit model (the inset of Figure 8e), showing that C_OCLP-4.5_ has the lowest R_ct_ (internal charge transfer resistance) and R_s_ (contact resistance with the electrolyte) among the three materials. That is, in the high-frequency region, the R_ct_ of C_OCLP-4.5_ is 0.042 Ω, lower than those of C_OCLP-3.5_ (0.152 Ω) and C_OCLP-5.0_ (0.183 Ω). The low R_s_ demonstrates that the electrolyte ions are readily transferred to the surface of the C_OCLP-4.5_ electrode [48]. Additionally, the linear curve of C_OCLP-4.5_ is almost vertical in the low-frequency region. The EIS results illustrate that the structure of C_OCLP-4.5_ is beneficial for charge transfer and the efficient diffusion of electrolyte ions. For supercapacitors, the cycling stability is a significant parameter to estimate their practical application. Figure 8f shows that C_OCLP-4.5_ retains 83% of its initial specific capacitance value after 5000 cycles at a current density of 5 A g^−1^. The surface morphology of C_OCLP-4.5_ after cycling was characterized by SEM, as shown in Figure 9. Compared with the C_OCLP-4.5,_ before (Figure 4b) shows that the pore structure of C_OCLP-4.5_ has some damage and collapses after 5000 cycles.

**Figure 10:**
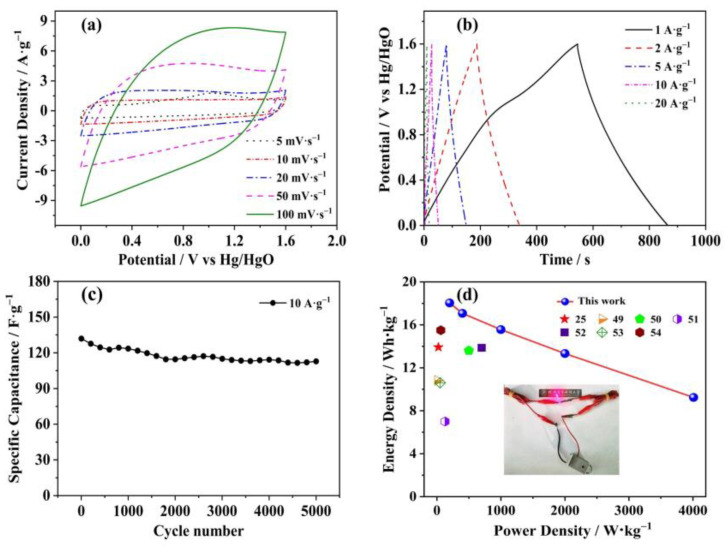
(**a**) CV curves at 5–100 mV·s^−1^. (**b**) GCD curves at 1–20 A·g^−1^. (**c**) Stable cyclic performance. (**d**) Ragone plots as compared to other studies and a supercapacitor device lighting up light-emitting diodes.

A symmetric supercapacitor was constructed using C_OCLP-4.5_ to evaluate its practical application. Figure 10a shows the CV curves for the symmetric supercapacitor at different scan rates. The curves maintained a quasi-rectangular shape at a scan rate of 50 mV·s^−1^. A slight deformation indicates that the electrochemical behavior of a symmetric supercapacitor is a combination of the EDLC and pseudocapacitance. Figure 10b shows that the GCD curves for the symmetric supercapacitor increased with an increasing current density from 1 to 20 A·g^−1^. Based on Equation (2), the specific capacitance of C_OCLP-4.5_ is 203 F·g^−1^ at 1 A·g^−1^ and its specific capacitance remains 150 F·g^−1^ at 10 A·g^−1^, demonstrating a good rate capability even at high current densities for the symmetric supercapacitor. Figure 10c shows the cycle stability curve at a current density of 10 A·g^−1^. It displays that the capacitance retention of the device is 84.0% after 5000 cycles, reflecting good cycling stability. Figure 10d indicates that the symmetric capacitor obtains an energy density of 18.04 Wh·kg^−^^1^ at a power density of 200.0 W·kg^−1^ based on Equations (3) and (4), significantly higher than those reported in recent years (13. [25], 10.83 [49], 13.60 [50], 7.00 [51], 13.86 [52], 10.60 [53], and 15.50 Wh·kg^−1^ [54]). Specifically, the symmetric supercapacitor device successfully powers up a light-emitting diode (the inset of Figure 10d). As shown in the video (see Supplementary Materials File S1), the light-emitting diode can last for a while. Obviously, the N/O-doped porous C_OCLPs_ are expected to be used in supercapacitors.

## 4. Conclusions

In this study, a network-structured organic crosslinked polymer was used as a carbon source to obtain N/O-doped porous C_OCLPs._ The results indicated that the C_OCLP-4.5_ obtained by optimizing the raw materials exhibited an excellent electrochemical performance. For instance, the specific capacitance of C_OCLP-4.5_ was as high as 522 F·g^−1^ at a current density of 0.5 A·g^−1^, and still exhibited 309 F·g^−1^ at 20 A·g^−1^ in a three-electrode system. Furthermore, the symmetric capacitor achieved an energy density of 18.04 Wh·kg^−1^ at a power density of 200.0 W·kg^−1^. The C_OCLPs_ benefitted from the net structure of organic crosslinked polymers to form hierarchical porous carbon and the pseudocapacitance introduced by heteroatoms. Therefore, the method for fabricating carbon material proposed in this study provides a new strategy for the development of electrode materials with high electrochemical performance.

## Figures and Tables

**Table 1:** Channel structure parameters of the C_OCLPs_.

Samples	Specific Surface Area (m^2^·g^−1^)			Pore Volume (cm^3^·g^−1^)		
	Total	Microporous	Mesoporous	Total	Microporous	Mesoporous
C_OCLP-3.5_	942	894	48	0.399	0.353	0.046
C_OCLP-4.5_	1589	1509	80	0.657	0.592	0.065
C_OCLP-5.0_	1102	1040	62	0.482	0.407	0.075

**Table 2:** Surface element content of the C_OCLPs_.

Samples	Element Content		
	Carbon (%)	Nitrogen (%)	Oxygen (%)
C_OCLP-3.5_	92.83	1.98	5.19
C_OCLP-4.5_	85.75	1.68	12.75
C_OCLP-5.0_	84.51	2.65	12.84

**Table 3:** Comparison of the specific capacitances of the C_OCLP-4.5_ electroactive material to recently reported carbonaceous materials.

Material	Electrolyte	Current Density (A·g^−1^)	Capacitance (F·g^−1^)	Reference
Grape marc	6 M KOH	0.5	446	[23]
Polyphosphazene	6 M KOH	0.5	438	[25]
Polypyrrole/Polythiophene	KOH	0.5	455	[41]
Cotton stalk	1 M H_2_SO_4_	0.2	338	[42]
L-tyrosine	KOH	0.3	512	[43]
Coal tar pitch	6 M KOH	0.5	298	[44]
CNTs@Gr-CNF	6 M KOH	0.25	521	[45]
CTAB	6 M KOH	1.0	241	[46]
3-aminophenol-formaldehyde resin	6 M KOH	0.5	381	[47]
Organic crosslinked polymer	6 M KOH	0.5	522	This work

## Data Availability

All data are available upon reasonable request.

## Supplementary Materials

The following supporting information can be downloaded at: https://www.mdpi.com/article/10.3390/nano12132186/s1, File S1: Lighting up LED lights video.

## References

[1] Hou J., Cao C., Idrees F., Ma X. (2015). Hierarchical Porous Nitrogen-Doped Carbon Nanosheets Derived from Silk for Ultrahigh-Capacity Battery Anodes and Supercapacitors. ACS Nano.

[2] Zou K., Deng Y., Chen J., Qian Y., Yang Y., Li Y., Chen G. (2018). Hierarchically porous nitrogen-doped carbon derived from the activation of agriculture waste by potassium hydroxide and urea for high-performance supercapacitors. J. Power Sources.

[3] Chmiola J., Yushin G., Dash R., Gogotsi Y. (2006). Effect of pore size and surface area of carbide derived carbons on specific capacitance. J. Power Sources.

[4] Wu Y., Cao J., Zhao X., Zhuang Q., Zhou Z., Huang Y., Wei X. (2020). High-performance electrode material for electric double-layer capacitor based on hydrothermal pre-treatment of lignin by ZnCl_2_. Appl. Surf. Sci..

[5] Li Z., Zhang L., Amirkhiz B.S., Tan X., Xu Z., Wang H., Olsen B.C., Holt C.M.B., Mitlin D. (2012). Carbonized Chicken Eggshell Membranes with 3D Architectures as High-Performance Electrode Materials for Supercapacitors. Adv. Energy Mater..

[6] Sun L., Tian C., Li M., Meng X., Wang L., Wang R., Yin J., Fu H. (2013). From coconut shell to porous graphene-like nanosheets for high-power supercapacitors. J. Mater. Chem. A.

[7] Wang H., Xu Z., Kohandehghan A., Li Z., Cui K., Tan X., Stephenson T.J., King ondu C.K., Holt C.M.B., Olsen B.C. (2013). Interconnected Carbon Nanosheets Derived from Hemp for Ultrafast Supercapacitors with High Energy. ACS Nano.

[8] Chang Y., Shi H., Yan X., Zhang G., Chen L. (2020). A ternary B, N, P-Doped carbon material with suppressed water splitting activity for high-energy aqueous supercapacitors. Carbon.

[9] Wang Y., Wang D., Li Z., Su Q., Wei S., Pang S., Zhao X., Liang L., Kang L., Cao S. (2022). Preparation of Boron/Sulfur-Codoped Porous Carbon Derived from Biological Wastes and Its Application in a Supercapacitor. Nanomaterials.

[10] Wang P., Ding X., Zhe R., Zhu T., Qing C., Liu Y., Wang H. (2022). Synchronous Defect and Interface Engineering of NiMoO_4_ Nanowire Arrays for High-Performance Supercapacitors. Nanomaterials.

[11] Han X., Tao K., Wang D., Han L. (2018). Design of a porous cobalt sulfide nanosheet array on Ni foam from zeolitic imidazolate frameworks as an advanced electrode for supercapacitors. Nanoscale.

[12] Khan S., Alkhedher M., Raza R., Ahmad M.A., Majid A., Din E.M.T.E. (2022). Electrochemical Investigation of PANI:PPy/AC and PANI:PEDOT/AC Composites as Electrode Materials in Supercapacitors. Polymers.

[13] Meng Q., Cai K., Chen Y., Chen L. (2017). Research progress on conducting polymer based supercapacitor electrode materials. Nano Energy.

[14] Shi L., Ye J., Lu H., Wang G., Lv J., Ning G. (2021). Flexible all-solid-state supercapacitors based on boron and nitrogen-doped carbon network anchored on carbon fiber cloth. Chem. Eng. J..

[15] Wu D., Cheng J., Wang T., Liu P., Yang L., Jia D. (2019). A Novel Porous N- and S-Self-Doped Carbon Derived from Chinese Rice Wine Lees as High-Performance Electrode Materials in a Supercapacitor. ACS Sustain. Chem. Eng..

[16] Lei W., Yang B., Sun Y., Xiao L., Tang D., Chen K., Sun J., Ke J., Zhuang Y. (2021). Self-sacrificial template synthesis of heteroatom doped porous biochar for enhanced electrochemical energy storage. J. Power Sources.

[17] Jiang W., Cai J., Pan J., Guo S., Sun Y., Li L., Liu X. (2019). Nitrogen-doped hierarchically ellipsoidal porous carbon derived from Al-based metal-organic framework with enhanced specific capacitance and rate capability for high performance supercapacitors. J. Power Sources.

[18] Lima R.M.A.P., Dos R.G.S., Thyrel M., AlcarazEspinoza J.J., Larsson S.H., de Oliveira H.P. (2022). Facile Synthesis of Sustainable Biomass-Derived Porous Biochars as Promising Electrode Materials for High-Performance Supercapacitor Applications. Nanomaterials.

[19] Alabadi A., Yang X., Dong Z., Li Z., Tan B. (2014). Nitrogen-doped activated carbons derived from a co-polymer for high supercapacitor performance. J. Mater. Chem. A.

[20] Park M., Ryu J., Kim Y., Cho J. (2014). Corn protein-derived nitrogen-doped carbon materials with oxygen-rich functional groups: A highly efficient electrocatalyst for all-vanadium redox flow batteries. Energy Environ. Sci..

[21] Paraknowitsch J.P., Thomas A., Antonietti M. (2010). A detailed view on the polycondensation of ionic liquid monomers towards nitrogen doped carbon materials. J. Mater. Chem..

[22] Inagaki M., Toyoda M., Soneda Y., Morishita T. (2018). Nitrogen-doped carbon materials. Carbon.

[23] Zhang J., Chen H., Bai J., Xu M., Luo C., Yang L., Bai L., Wei D., Wang W., Yang H. (2021). N-doped hierarchically porous carbon derived from grape marcs for high-performance supercapacitors. J. Alloys Compd..

[24] Mohd Abdah M.A.A., Azman N.H.N., Kulandaivalu S., Abdul Rahman N., Abdullah A.H., Sulaiman Y. (2019). Potentiostatic deposition of poly (3, 4-ethylenedioxythiophene) and manganese oxide on porous functionalised carbon fibers as an advanced electrode for asymmetric supercapacitor. J. Power Sources.

[25] Zou W., Zhang S., Abbas Y., Liu W., Zhang Y., Wu Z., Xu B. (2020). Structurally designed heterochain polymer derived porous carbons with high surface area for high-performance supercapacitors. Appl. Surf. Sci..

[26] Chen Y., Liu F., Qiu F., Lu C., Kang J., Zhao D., Han S., Zhuang X. (2018). Cobalt-Doped Porous Carbon Nanosheets Derived from 2D Hypercrosslinked Polymer with CoN_4_ for High Performance Electrochemical Capacitors. Polymers.

[27] Chen D., Xu F., Sun L., Xia Y., Wei S., Zhang H. (2020). Preparation and thermal property of PEG based composite phase change material. New Chenical Mater..

[28] Chen C., Liu W., Wang H., Peng K. (2015). Synthesis and performances of novel solid–solid phase change materials with hexahydroxy compounds for thermal energy storage. Appl. Energy.

[29] Wang Y., Zhang M., Dai Y., Wang H., Zhang H., Wang Q., Hou W., Yan H., Li W., Zheng J. (2019). Nitrogen and phosphorus co-doped silkworm-cocoon-based self-activated porous carbon for high performance supercapacitors. J. Power Sources.

[30] Zhipeng Q., Yesheng W., Xu B., Tong Z., Jin Z., Jinping Z., Zhichao M., Weiming Y., Peng F., Shuping Z. (2018). Biochar-based carbons with hierarchical micro-meso-macro porosity for high rate and long cycle life supercapacitors. J. Power Sources.

[31] Kong L.N., Yang W., Su L., Hao S.G., Shao G.J., Qin X.J. (2019). Nitrogen-doped 3D web-like interconnected porous carbon prepared by a simple method for supercapacitors. Ionics.

[32] Boujibar O., Ghamouss F., Ghosh A., Achak O., Chafik T. (2019). Activated carbon with exceptionally high surface area and tailored nanoporosity obtained from natural anthracite and its use in supercapacitors. J. Power Sources.

[33] Hu J., He W., Qiu S., Xu W., Mai Y., Guo F. (2019). Nitrogen-doped hierarchical porous carbons prepared via freeze-drying assisted carbonization for high-performance supercapacitors. Appl. Surf. Sci..

[34] Yiju L., Guiling W., Tong W., Zhuangjun F., Peng Y. (2016). Nitrogen and sulfur co-doped porous carbon nanosheets derived from willow catkin for supercapacitors. Nano Energy.

[35] Jia H., Zhang H., Wan S., Sun J., Xie X., Sun L. (2019). Preparation of nitrogen-doped porous carbon via adsorption-doping for highly efficient energy storage. J. Power Sources.

[36] Javaid A., Irfan M. (2018). Multifunctional structural supercapacitors based on graphene nanoplatelets/carbon aerogel composite coated carbon fiber electrodes. Mater. Res. Express.

[37] Li C., Wu W., Wang P., Zhou W., Wang J., Chen Y., Fu L., Zhu Y., Wu Y., Huang W. (2019). Fabricating an Aqueous Symmetric Supercapacitor with a Stable High Working Voltage of 2 V by Using an Alkaline-Acidic Electrolyte. Adv. Sci..

[38] Li S., Fan Z. (2019). Nitrogen-doped carbon mesh from pyrolysis of cotton in ammonia as binder-free electrodes of supercapacitors. Microporous Mesoporous Mater..

[39] Méndez-Morales T., Ganfoud N., Li Z., Haefele M., Rotenberg B., Salanne M. (2019). Performance of microporous carbon electrodes for supercapacitors: Comparing graphene with disordered materials. Energy Storage Mater..

[40] Huo S., Liu M., Wu L., Liu M., Xu M., Ni W., Yan Y. (2019). Synthesis of ultrathin and hierarchically porous carbon nanosheets based on interlayer-confined inorganic/organic coordination for high performance supercapacitors. J. Power Sources.

[41] Lu Y., Liang J., Deng S., He Q., Deng S., Hu Y., Wang D. (2019). Hypercrosslinked polymers enabled micropore-dominant N, S Co-Doped porous carbon for ultrafast electron/ion transport supercapacitors. Nano Energy.

[42] Cheng J., Hu S., Sun G., Kang K., Zhu M., Geng Z. (2021). Comparison of activated carbons prepared by one-step and two-step chemical activation process based on cotton stalk for supercapacitors application. Energy.

[43] Wang M., Han K., Qi J., Teng Z., Zhang J., Li M. (2022). Study on performance and charging dynamics of N/O codoped layered porous carbons derived from L-tyrosine for supercapacitors. Appl. Surf. Sci..

[44] Yang X., Zhao S., Zhang Z., Chi Y., Yang C., Wang C., Zhen Y., Wang D., Fu F., Chi R. (2022). Pore structure regulation of hierarchical porous carbon derived from coal tar pitch via pre-oxidation strategy for high-performance supercapacitor. J. Colloid Interface Sci..

[45] Tolendra K., Duy T.T., Dinh C.N., Nam H.K., Kin-tak L., Joong H.L. (2020). Ternary graphene-carbon nanofibers-carbon nanotubes structure for hybrid supercapacitor. Chem. Eng. J..

[46] Zhang F., Zong S., Zhang Y., Lv H., Liu X., Du J., Chen A. (2021). Preparation of hollow mesoporous carbon spheres by pyrolysis-deposition using surfactant as carbon precursor. J. Power Sources.

[47] Juan D., Yue Z., Haixia W., Senlin H., Aibing C. (2020). N-doped hollow mesoporous carbon spheres by improved dissolution-capture for supercapacitor. Carbon.

[48] Hongmei H., Li M., Shenna F., Mengyu G., Liangqing H., Huanhuan Z., Fei X., Minghang J. (2019). Fabrication of 3D ordered honeycomb-like nitrogen-doped carbon/PANI composite for high-performance supercapacitors. Appl. Surf. Sci..

[49] Zhou Z., Cao J., Wu Y., Zhuang Q., Zhao X., Wei Y., Bai H. (2021). Waste sugar solution polymer-derived N-doped carbon spheres with an ultrahigh specific surface area for superior performance supercapacitors. Int. J. Hydrogen Energy.

[50] He W., Haitao N., Hongjie W., Wenyu W., Xin J., Hongxia W., Hua Z., Tong L. (2021). Micro-meso porous structured carbon nanofibers with ultra-high surface area and large supercapacitor electrode capacitance. J. Power Sources.

[51] Man W., Juan Y., Siyu L., Muzi L., Chao H., Jieshan Q. (2020). Nitrogen-doped hierarchically porous carbon nanosheets derived from polymer/graphene oxide hydrogels for high-performance supercapacitors. J. Colloid Interf. Sci..

[52] Li J., Zou Y., Xiang C., Xu F., Sun L., Li B., Zhang J. (2021). Osmanthus fragrans-derived N-doped porous carbon for supercapacitor applications. J. Energy Storage.

[53] Liu H., Song H., Hou W., Chang Y., Zhang Y., Li Y., Zhao Y., Han G. (2021). Coal tar pitch-based hierarchical porous carbons prepared in molten salt for supercapacitors. Mater. Chem Phys..

[54] Zhang Y., Wu C., Dai S., Liu L., Zhang H., Shen W., Sun W., Ming L.C. (2022). Rationally tuning ratio of micro- to meso-pores of biomass-derived ultrathin carbon sheets toward supercapacitors with high energy and high power density. J. Colloid Interf. Sci..

