# Supplementary material for: Organic Crosslinked Polymer-Derived N/O-Doped Porous Carbons for High-Performance Supercapacitor
Source: Nanomaterials (Basel). 2022 Jun 25;12(13):2186. doi: 10.3390/nano12132186 (PMC9268302; doi:10.3390/nano12132186)
Supplement: Supplementary file 1 [file nanomaterials-12-02186-s001.zip › nanomaterials-1774652-supplementary materials.pdf]

supplementary materials

Organic Crosslinked Polymer-Derived N/O-Doped Porous Carbons for High-Performance Supercapacitor

Jianhao Lao [1,†], Yao Lu [1,†], Songwen Fang [1], Fen Xu [1,*], Lixian Sun [1,*], Yu Wang [1], Tianhao Zhou [1], Lumin Liao [1,2], Yanxun Guan [1,2], Xueying Wei [3,*], Chenchen Zhang [1], Yukai Yang [1], Yongpeng Xia [1], Yumei Luo [1], Yongjin Zou [1], Hailiang Chu [1], Huanzhi Zhang [1], Yong Luo [1] and Yanling Zhu [1]

[1] Guangxi Key Laboratory of Information Materials, Guangxi Collaborative Innovation Center for Structure and Properties for New Energy and Materials, School of Material Science and Engineering, Guilin University of Electronic Technology, Guilin 541004, China; ljh408888@163.com (J.L.); 18677367876@163.com (Y.L.); 1810201010@mails.guet.edu.cn (S.F.); ywang506x@163.com (Y.W.); zhoutianhao233@gmail.com (T.Z.); llm904691049@163.com (L.L.); gyx112405@163.com (Y.G.); Zhang_linba_3760@163.com (C.Z.); yangyukai0530@gmail.com (Y.Y.); ypxia@guet.edu.cn (Y.X.); luoym@guet.edu.cn (Y.L.); zouy@guet.edu.cn (Y.Z.); chuhailiang@guet.edu.cn (H.C.); zhanghuanzhi@guet.edu.cn (H.Z.); 15620323072@139.com (Y.L.); zyl9352021@163.com (Y.Z.)

[2] School of Electronic Engineering and Automation, Guilin University of Electronic Technology, Guilin 541004, China

[3] School of Architecture and Transportation Engineering, Guilin University of Electronic Technology, Guilin 541004, China

* Correspondence: xufen@guet.edu.cn (F.X.); sunlx@guet.edu.cn (L.S.); http510@guet.edu.cn (X.W.)

† These authors contributed equally to this work.

File 1: Lighting up LED lights video, as follows.

Lighting up LED lights video--Organic Cross-Linked Polymer-Derived NO-Doped Porous Carbons for High-Performance Supercapacitor..mp4
